# Hemispheric dominance underlying the neural substrate for learned vocalizations develops with experience

**DOI:** 10.1038/srep11359

**Published:** 2015-06-22

**Authors:** Napim Chirathivat, Sahitya C. Raja, Sharon M. H. Gobes

**Affiliations:** 1 Neuroscience Program, Wellesley College, 106 Central Street, Wellesley, MA 02481.

## Abstract

Many aspects of song learning in songbirds resemble characteristics of speech acquisition in humans. Genetic, anatomical and behavioural parallels have most recently been extended with demonstrated similarities in hemispheric dominance between humans and songbirds: the avian higher order auditory cortex is left-lateralized for processing song memories in juvenile zebra finches that already have formed a memory of their fathers’ song, just like Wernicke’s area in the left hemisphere of the human brain is dominant for speech perception. However, it is unclear if hemispheric specialization is due to pre-existing functional asymmetry or the result of learning itself. Here we show that in juvenile male and female zebra finches that had never heard an adult song before, neuronal activation after initial exposure to a conspecific song is bilateral. Thus, like in humans, hemispheric dominance develops with vocal proficiency. A left-lateralized functional system that develops through auditory-vocal learning may be an evolutionary adaptation that could increase the efficiency of transferring information within one hemisphere, benefiting the production and perception of learned communication signals.

The parallels between speech acquisition in humans and song learning in songbirds are numerous^1^,^2^,^3^,^4^,^5^. In both cases there is a sensitive period for auditory learning during which the infants or young birds are exposed to and memorize the sounds of adult conspecifics. In zebra finches (*Taeniopygia guttata*), a memory of the song of the tutor is usually formed between 25–35 days post hatching (dph)^6^, but the duration of the sensitive period is dependent on model availability; even after 65 days post hatching, birds will successfully copy song from an adult male introduced to their cage^7^,^8^. Under normal circumstances song becomes ‘crystallized’ and does not change anymore in adulthood (>120 dph), but birds with limited experience with song models can still modify their songs beyond the conventional sensitive period for song learning^9^,^10^,^11^. Similar to timing of model availability in birds described above, a second language can replace the first language in adopted children even when exposure to the second language did not happen until 3–8 years of age^12^. Thus, auditory and vocal learning phases overlap in humans and songbirds and the end of the sensitive phase for auditory learning depends on model availability. In contrast, learning to produce vocalizations progresses through distinct phases: from ‘cooing’ to ‘babbling’ in human infants and ‘subsong’ to ‘plastic song’ in songbirds^13^,^14^.

In humans, it is well known that regions within the superior temporal gyrus and sulcus, including Wernicke’s area, are involved in speech perception. The songbird analogue of the mammalian higher auditory cortex is localized in the auditory lobule of the avian forebrain (see Fig. 1). The auditory lobule consists of the caudo-medial Nidopallium (NCM) and Mesopallium (CMM), analogous to regions in Superior Temporal cortex in humans, including Wernicke’s Area, and the primary auditory area, Field L^2^. Results of several studies indicate that these regions are (part of) the neural substrate for auditory perception and recognition memory^15^,^16^,^17^. Converging evidence suggests that the NCM is also involved in acquisition, processing, or recognition of the memory of the tutor song specifically^18^,^19^,^20^,^21^,^22^, which is acquired early in life, with parallel or distributed loci for the representation of this memory existing within the song system^23^,^24^.

Brain regions involved in human speech perception and production are functionally lateralized^25^,^26^. If such lateralization is related to successful auditory-vocal learning, Moorman and colleagues (2012) hypothesized that brain activation for auditory memory would be lateralized in juvenile songbirds that are in the middle of their song-learning period. When juvenile males were re-exposed to the song of their tutor, there was predominant activation in the left NCM, similar to that seen in humans^27^. Auditory memory was left lateralized (measured as a positive lateralization ratio) for males that already had imitated characteristics of their tutor’s song, but not for birds with less similarity to their tutors’ songs^27^. Activation in the pre-motor nucleus HVC (used as a proper noun), which forms part of a circuit equivalent to the ventral prefrontal cortex (including Broca’s area) in humans^28^, was dominant in the left hemisphere independent of the auditory stimulus to which the birds were exposed^27^. This suggests that lateralization of the auditory-vocal system may be necessary for successful vocal learning. During language learning in humans, left-lateralization becomes more significant with age and with increasing language proficiency^29^,^30^,^31^,^32^; when proficiency of a newly learned language is poor, there is a largely bilateral activation pattern^33^ and language impairments (developmental dysphasias) have been linked to a lack of lateralization in the core language areas^34^. If pronounced left-lateralization of the neural substrate for auditory processing is characteristic for successful development of acquired vocal skills, the question arises whether left-hemispheric dominance is absent at the onset of learning in other vocal learning species, such as songbirds. Establishing a parallel in lateralization of brain regions, or the absence thereof at the onset of the learning process, is key in relating findings from the songbird model to humans.

Is hemispheric dominance underlying the neural substrate for vocal learning innate, or does it develop with skill acquisition? Pre-natal experience with language in human infants may shape lateralization of the neural substrates underlying speech perception^35^,^36^,^37^ and, therefore, the relationship between pre-existing functional asymmetry and lateralization resulting from learning is unclear. Similarly, the results of the study by Moorman *et al.* (2012) do not allow us to distinguish between the following hypotheses: 1) some birds were already left-dominant for neuronal activity in NCM when they *first* listened to the song of their tutor and therefore became better vocal imitators (pre-existing functional asymmetry), 2) in birds that sang accurate imitations while practicing their motor skills, the neural substrate for song memory became more left-lateralized through experience (lateralization resulting from proficiency), or 3) a small pre-existing bias in processing song with the left NCM became more pronounced in good learners (pre-existing functional asymmetry enhanced by proficiency). Here, we conducted similar experiments in previously isolated male and female zebra finches to distinguish between these hypotheses. Female zebra finches do not learn to produce a song, but develop a preference for the song of their father^38^,^39^. Comparing male and female zebra finches can thus reveal the neural mechanisms of auditory memory independent of song-production learning^40^,^41^,^42^. We hypothesized that if song acquisition parallels speech learning in humans, the neural response to song should initially be bilateral in the NCM of birds that have no prior experience with the vocalizations produced by adult conspecifics.

## Results

**Figure 2:**
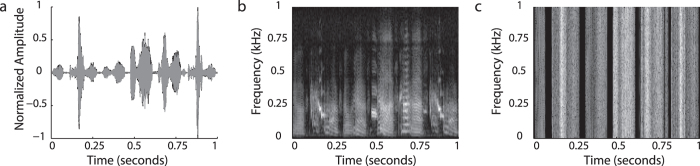
Auditory stimuli for playback experiments. (**a**) An overlay of the amplitude plots of the tutor stimulus (black) and white noise control stimulus (grey), showing matching amplitude and temporal envelop of both stimuli. Spectrograms of the song (**b**) and control stimulus (**c**). Note the differences in spectro-temporal characteristics.

To control the auditory environment and limit exposure to normal adult songs early in life, juvenile zebra finches were separated from their father when they were 7–10 days old (when hearing thresholds are still elevated compared to adults) and raised by their mother (‘isolates’)^43^. The juvenile males were put into individual sound-isolated boxes at ~37 days post hatching (dph), which is when male juveniles start to produce ‘subsong’. Experiments were performed between 45–98 dph, a period in development during which molecular responses are inducible in normally reared male and female zebra finches^44^,^45^. We exposed juvenile birds to one of three auditory stimuli: a 30-min playback of a song from an adult conspecific (Song), a rhythmic white-noise stimulus for which the RMS-amplitude and temporal envelope were exactly matched to the song stimuli in the experimental group (Noi; see Fig. 2 and Park & Clayton^46^) or to silence (Sil). Thus, the only difference between Song and Noise groups was the species-specific spectral and temporal characteristics present in the song-stimulus but not in the noise control stimulus (Fig. 2). The birds were sacrificed 30 min after stimulus exposure, which is optimal to induce protein expression in the auditory regions^47^, and their brains processed immunocytochemically for Zenk (the protein product of the immediate early gene *ZENK,* an acronym for *zif-268, egr-1, ngf-Ia* and *krox-24*), a marker for neuronal activation^48^(for review, see: Mello *et al*., 2004^49^).

### Differential modulation of the subdivisions of the avian auditory lobule

**Figure 3:**
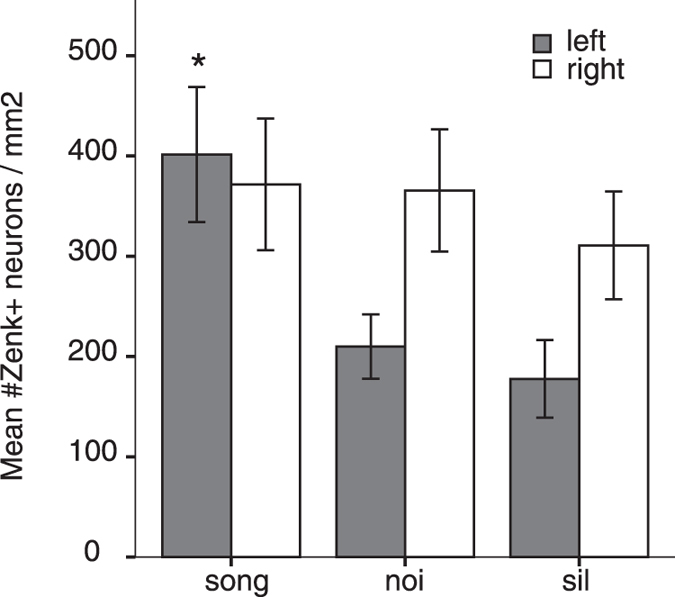
Zenk expression in the left and right NCM of juvenile zebra finches. Mean number of Zenk-immunopositive neurons per square millimeter in the NCM is shown for the left (grey) and right (white) hemisphere. The mean activation level is higher in the NCM of zebra finches that were exposed to tutor song than to noise or silence, in the left hemisphere only (indicated with asterisk, see Results for statistics). Error bars represent the SEM.

We quantified the number of Zenk-immunopositive neurons bilaterally in the NCM (Fig. 3) and CMM to investigate stimulus-specific lateralization. To examine the effects of auditory stimulus on the Zenk response in the different brain regions in both sexes, a repeated measures ANOVA with Hemisphere (left or right) as within-subjects factor and Stimulus (song, noise or silence) and Sex (male or female subjects) as between-subject factors was conducted for NCM as well as CMM. This analysis showed that there was a significant effect in NCM of Hemisphere (F_1,25_ = 11.22, p = 0.003) and a significant interaction between Hemisphere and Stimulus (F_2,25_ = 5.96, p = 0.008) but no interactions between Hemisphere and Sex (F_1,25_ = 0.001, n.s.) or Hemisphere, Stimulus and Sex (F_2,25_ = 2.83, n.s.). This effect was due to a stimulus-dependent response in the left NCM only (Song > Sil, p = 0.006, Song > Noi, p = 0.016 Bonferronni corrected post-hoc test, Fig. 3). In the right NCM, there were no stimulus-dependent responses; activation to Song or Noi was not significantly different than the background (or default) activation seen in the Sil group (p > 0.05 Bonferronni corrected post-hoc test). A similar analysis was performed in CMM, the results of which showed no significant effects of Hemisphere or Stimulus on neuronal activation (p > 0.05 for all factors and interactions between factors) but a significant effect of Sex (F_1,21_ = 5.30, p = 0.032; no interactions with other factors).

Some males had produced songs in the hour before they were sacrificed. We measured the total duration (in seconds) of songs produced by these birds. There were no significant correlations between song production and neuronal activation in any of the regions investigated (Pearson’s correlation coefficient; p > 0.05 for NCM and CMM). We investigated whether the age at which the playback experiment was performed affected the levels of Zenk expression in each of the four regions and in each stimulus group independently; no significant correlations were found (Pearson’s correlation coefficient with Bonferroni adjusted α for testing in four regions: p = n.s. for all regions and all stimulus groups; before adjustments for multiple testing, right CMM in Song group: r = 0.65, N = 13, p = 0.016; left CMM in Noi group: r = 0.67, N = 9, p = 0.047; all other regions p > 0.05).

### The first exposure to song: neuronal activation in the NCM is bilateral

Because of the stimulus-dependent hemispheric differences in neuronal activation in NCM described in the previous paragraph, we calculated the lateralization ratio ([L − R]/[L + R]) for each bird. This lateralization ratio normalizes individual lateralization levels in response to each stimulus and is not influenced by differences in absolute neuronal activation.

**Figure 4:**
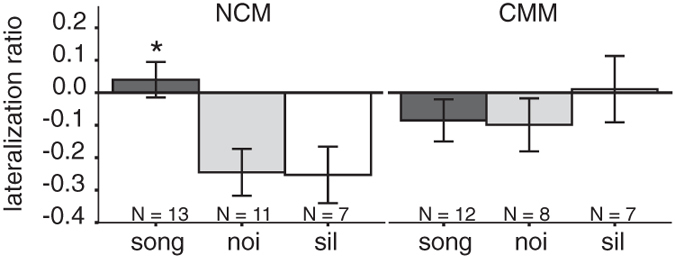
Lateralization ratios in the NCM and CMM after exposure to song, rhythmic white noise or silence. Lateralization ratios ([L − R]/[L + R]) were calculated for each subject from the number of Zenk-immunopositive cells per square millimeter. In the NCM, the data show equal bilateral activation in response to a song (mean lateralization ratio ± SEM; dark grey bar). In white-noise exposed birds (grey bar) and birds kept in silence (white bar), the lateralization ratio is negative, indicating stronger activation of the right hemisphere. Asterisk indicates a significant difference between the groups. For CMM, there was bilateral activation independent of stimulus condition.

ANOVA confirmed a significant effect of Stimulus on lateralization ratios in the NCM (Fig. 4; F_2,31_ = 6.20, p = 0.007; Bonferroni corrected post-hoc tests: Song >Noi p = 0.008, Song >Sil p = 0.019). There were no significant effects of Sex (F_1,31_ = 0.00, p = n.s.) or between Stimulus*Sex (F_2,31_ = 2.78, p = n.s.). Thus, in birds that do not have previous experience with a song from an adult conspecific, there was a bilateral neuronal response in the NCM when first exposed to song (Fig. 4; mean lateralization ratio = 0.04; t = 0.738, p = n.s., n = 13). The lateralization ratios for the rhythmic noise and silence groups were significantly different from zero in the NCM, demonstrating right-hemispheric dominance when birds are not exposed to a model song (Noi: mean lateralization ratio = −0.24, t = −3.39, p = 0.007, n = 11; Sil: mean lateralization ratio = −0.25, t = −2.91, p = 0.027, n = 7). For comparison (see Fig. 4a in Moorman *et al.* 2012), lateralization ratios for birds that have already learned song and were re-exposed to their tutor song, ranged between −0.2 and 0.8 depending on learning proficiency^27^.

Lateralization ratios for CMM are also shown in Fig. 4. In the CMM, lateralization ratios indicated bilateral activation independent of stimulus in both males and females (p > 0.05 for all factors and stimuli).

## Discussion

These findings establish that neuronal activation in isolated juvenile songbirds is bilateral in the NCM, a region functionally analogous to Wernicke’s area in the mammalian brain, when they are first exposed to a model song. In contrast, neuronal activation in the control groups, whether spontaneous (no stimulus) or evoked by a rhythmic white-noise stimulus, is right-dominant. This pattern of differential activation is due to modulation of the neuronal response in the left hemisphere only, with song inducing significantly stronger response than silence. The default processing state of the NCM in animals with no prior experience with song is thus one in which neuronal activation can be induced by song in the left hemisphere only, but not in the right hemisphere due to higher background activation. Thus, an innate bias to process song with the left hemisphere (this article) results in balanced neuronal activation between left and right hemispheres when birds are *first* exposed to song. Taken together with the results from Moorman *et al.* (2012) showing left-lateralization in juveniles that had already learned song^27^, the current results support the hypothesis that left-*dominance* of the neural substrate for song memory is not innate but develops with successful song imitation. Thus, these results confirm hypothesis 3 (outlined in the introduction): a pre-existing functional asymmetry in inducible neuronal activation becomes enhanced by proficiency. Whether hemispheric dominance arises due to an increase in activation in the left hemisphere, or a decrease in baseline activity levels in the right hemisphere remains to be determined.

Consistent with the current results, a previous study that measured absolute response magnitudes (ARMs) in the NCM of adult male and female zebra finches also showed a lack of lateralization in birds that had no experience with song^50^. However, ARMs to both songs and long calls were stronger in the right hemisphere in birds that had previous experience with either a tutor song or their own (isolate) song than in birds that had never heard song before^50^. In contrast, there is left-hemispheric dominance in IEG expression in birds exposed to their tutor’s song but not in birds exposed to a novel song^27^. Thus, the right-hemispheric dominance in experienced birds that was reported in the study by Phan and Vicario (2010) could be due to differences in auditory processing of the stimulus related to memory: an unfamiliar song^50^ vs. the tutor song^27^ or the method used (electrophysiology vs. immediate early gene expression). Alternatively, this could be due to the behavioural state of the subject: anesthetized^50^ vs. awake behaving^27^ birds.

It is relevant to note that ~40% of Zenk-positive neurons in the NCM are GABAergic^51^. Pharmacological application of GABA antagonists results in a more phasic and synchronized response pattern of auditory units in the NCM and abolishes sustained activity between the song syllables^52^. A role for inhibitory circuits in shaping auditory selectivity has been shown in the NCM of European starlings (*Sturnus vulgaris*) that had learned to associate certain song stimuli in a go/no go task^53^. We could interpret the left-hemispheric dominance for tutor song that develops with successful song imitation, as the result of stronger inhibition and thus higher selectivity for learned song. A pre-existing bias of the left NCM to process song would then allow the left NCM to become dominant for processing auditory memories, such as the memory of the tutor’s song or the bird’s own song, through strengthening and activation of inhibitory (and possibly disinhibitory) local circuits.

The presence of balanced bilateral activation in response to song in the current study could be explained by a hemispheric difference in modulation of the auditory response: in the left hemisphere neuronal activation was modulated differentially by the auditory stimuli, whereas in the right hemisphere there was no significant modulation (Fig. 3). This suggestion is also consistent with the behavioural preference for the bird’s own song, which can be abolished by blocking the production of estradiol in the left NCM only, but is not affected by the same treatment in the right NCM^54^. Lesions to the left ascending auditory pathway (nucleus Ovoidalis) have similar consequences on song discrimination in a go/no-go test, disrupting song discrimination when the BOS and a cage-mate’s song are used as stimuli whereas the right hemisphere seems to be involved in discriminating the subtle differences in harmonic profile of songs^55^. Thus, the right NCM seems to be dominant for perception of spectro-temporal characteristics of song and the left NCM for perception and recognition of familiar songs.

What is the neuronal mechanism through which the NCM becomes lateralized? In males that have no prior experience with a model song, the ERP, BOLD and IEG response lack a stimulus-specific response pattern^41^,^42^. Here, we show that the *left-dominant* IEG response typical for normally reared male juveniles^27^ is also absent in previously isolated birds. In juveniles that are raised with a song tutor, there is left-sided dominance for memorized song and bilateral processing of novel song^27^. Together, the results show that initial auditory processing is bilateral, and that the neural substrate for memorized song becomes left-dominant when vocal imitation becomes more accurate.

There is a parallel with human speech acquisition: functional specialization of the left-hemisphere for language becomes more pronounced with increasing complexity of language skills^32^. In addition, in bilingual toddlers, there is left lateralization for the dominant language and bilateral activation for the non-dominant language, indicating that left-lateralization develops with language proficiency similarly to song learning in songbirds^30^. Development of lateralization of the neural substrate for language may begin very early in development, possibly prenatally. In sleeping human neonates, there was a left-lateralized bias for processing forward versus backward speech^56^. Functional MRI experiments in 3-month old infants revealed left-hemispheric processing of auditory stimuli (forward and backward speech) in all infants (awake or asleep), whereas there was bilateral activation that was greater for backward speech versus forward speech in the posterior part of the superior temporal sulci only in awake infants^57^. Interestingly, language impairments such as developmental dysphasia, autism spectrum disorder, speech delay, stuttering and dyslexia, have all been linked to atypical or lack of lateralization (see^34^).

In conclusion, in juvenile songbirds with limited experience with song, there is balanced neuronal activation when first exposed to adult conspecific vocalizations; the left-dominance of brain regions that is characteristic of complex auditory-vocal skills is therefore likely a developmentally- and possibly evolutionarily-acquired trait.

## Materials & Methods

### Subjects

34 male and female juvenile zebra finches were reared in the animal facility of Wellesley College. Birds were maintained on a 16:8 light:dark cycle, lights on at 10:00 AM. All birds were kept in breeding cages with their parents and siblings until 7−11 days post-hatching (dph). At 9.7 dph (+/−0.25 SEM), the juveniles and their mother were separated from the father and transferred to a different room equipped with acoustically isolated cages each holding a single clutch. At 37 dph (+/−1 SEM; range 34–53 dph), juvenile males were transferred into individual, acoustically isolated holding cages. At 46 dph (+/−3 SEM; range 37–64 dph), juvenile females were also separated from their mother into individual, acoustically isolated holding cages. All experiments were performed during the equivalent of the plastic song stage in normally reared birds (~40–100 dph) (Immelmann 1969; Johnson *et al.* 2002). Mean age at the day of the experiment was 69 dph (+/−2.7 SEM; range 45–98 dph). Experimental procedures were in accordance with US law and approved by the Institutional Animal Care and Use Committee of Wellesley College (IACUC #1106).

### Experimental procedures

One day prior to stimulus exposure, the birds were transferred to a sound-proof chamber equipped with a microphone and a speaker. On the day of the experiment, the lights were manually turned on for the duration of stimulus exposure. Stimulus presentation started between 10:00 AM and 11:00 AM and lasted 30 min. The birds were sacrificed 30 min after the end of the last stimulus presentation. The birds were kept in darkness during the 30 min post-stimulus period to stop them from vocalizing, and thereby to prevent their own vocalizations from evoking molecular neuronal activation. Subjects were exposed to one of the three treatments: a recording of the song of an adult zebra finch male (Song), a rhythmic white-noise stimulus (Noi) with the same temporal envelope as song and of equal amplitude and duration (Fig. 2) or silence (Sil). Thus, the only difference between the Song and Noi is that the experimental group heard a stimulus that contains species-specific spectrotemporal characteristics. Each 30 min stimulus consisted of one-minute loops in which 15 seconds of sound was followed by 45 seconds of silence. The stimulus songs were broadcast through a speaker and Windows Media Player controlled the sound pressure level at 65 dB mean SPL at 30 cm from the speaker. Sound recordings were made throughout the experiment to ensure that birds were awake during stimulus presentation, and to monitor vocal behavior during stimulus exposure.

### Tissue collection

One hour after stimulus onset, the experimental subjects were anesthetized with 0.03 mL Natriumpentobarbital (intramuscular) (Fatal Plus, Vortech Pharmaceuticals, Dearborn, MI) and subsequently perfused with phosphate buffer (PB, pH 7.4) containing 0.2% heparin, followed by fixation with 2% paraformaldehyde and 0.075% glutaraldehyde in PB. Whole brains were dissected out, separated by hemisphere and post-fixed at 4 °C in 2% paraformaldehyde and 0.075% glutaraldehyde in PB for 4 hours. Parasaggital sections (50 um) were made on a vibratome and stored in PB overnight at 4 °C or in cryoprotectant at –18 °C.

### Immunocytochemistry

Sections were rinsed three times in PB (5 min. each rinse) and incubated in H_2_O_2_ (0.03%) for 8 minutes. Sections were rinsed three times with PBS and 0.01% BSA-c (acetylated albumin; BSA-c, Aurion, Wageningen, the Netherlands) for 10 minutes each and incubated with 5% normal goat serum (NGS) in 0.01% BSA-c for 30 minutes. Afterwards, sections were rinsed three times in PBS and 0.01% BSA-c for 5 minutes and incubated with primary polyclonal rabbit antiserum (Santa Cruz Biotechnology, Santa Cruz, CA; Cat. No. sc-189, 1:1,000) raised against the carboxy-terminus of mouse egr-1 (sequence STGLSDMTATFSPRTIEIC; see^47^) and 0.01% BSA-c overnight. Sections were rinsed again in PBS 10 times for 5 minutes, incubated with biotinylated goat anti-rabbit (IgG, dilution 1:500, Vector Laboratories, Burlingame, CA), for 1 hour (room temperature [RT]), and rinsed three times for 15 minutes in PBS. Afterwards, sections were incubated (RT) with ABC (avidin-biotinylated enzyme complex, Vector Elite Kit, Vector Laboratories) and rinsed in PBS twice for 5 minutes. Finally, sections were incubated in diaminobenzidine medium with 0.034% H_2_O_2_ for 4 minutes (RT). The reaction was stopped in distilled water. Sections were then rinsed in PBS, mounted on slides, dehydrated, and embedded in DPX (RT). Birds of different experimental groups were run in parallel with one another on the same well plate and controls were performed for which the primary or secondary antibodies were omitted. All procedures were performed cold (4 °C) unless otherwise specified.

### Image analysis

Quantification of Zenk-immunopositive cells was performed for NCM and CMM on 422 × 562 um images of three sections at the lateral position (between 600 and 1000 mm from the midline^18^,^21^,^58^,^59^). For the NCM, a counting frame was placed at the extreme caudal pole of the nidopallium^59^. For the CMM, the frame was placed adjacent to the ventricle and the lamina mesopallialis. Distance from the midline was assessed by calculating the number of serial sections and this location was verified using the atlas of Vates *et al.* (1996)^60^, an unpublished atlas of the zebra finch brain by A. M. den Boer-Visser (which was also used in previous studies^40^,^59^) and a stereotaxic atlas that is available online^61^. Digital photographs were taken using a SPOT Insight 2 Mp camera (SPOT Imaging Solutions) and the QCapture 2.9 program (Quantitative Imaging Corporation) on a Nikon Eclipse 50i (Nikon Instruments) with 20x objective. Image analysis was carried out with a PC-based system equipped with Image J (NIH, Bethesda, MD)^62^. Counts of three sections per region per animal were averaged for further statistical analysis. Image analysis was performed ‘blind’ as to the experimental history of the subject.

### Statistical analysis

Levels of Zenk expression were not normally distributed in three regions in the Song group, 2 regions in the Noise group and 2 regions in the Silence group (Kolmogorov-Smirnov test for normality, p < 0.05); data were natural-log transformed for further statistical analysis. A repeated measures ANOVA with Hemisphere (left or right) as within-subjects factor and Stimulus (song, noise or silence) and Sex (male or female subjects) as between-subject factors was conducted for NCM as well as CMM to examine the effects of playback stimulus on the levels of neuronal activation (measured as the number of Zenk+ neurons) in the different brain regions in both sexes (Bonferroni adjusted α  =  0.025 for performing repeated measures ANOVA on both NCM and CMM). A lateralization ratio for NCM and for CMM was calculated by dividing the difference in Zenk expression levels between the two hemispheres by the total amount of Zenk expression of the two hemispheres: [L − R]/[L + R]. To test for lateralization effects in each stimulus group, we performed an ANOVA on the lateralization ratios followed by post hoc paired t-tests with Bonferroni corrections for multiple testing. One-sample t-tests were performed to investigate whether lateralization ratios were significantly different from zero (bilateral). Pearson’s correlation coefficient with Bonferroni correction for multiple testing was used to examine correlations between Zenk expression levels and the age at which the experiment was performed, and with the amount of songs produced by the birds. Data were analyzed using SPSS 21.0.0 (IBM Corporation).

## Additional Information

**How to cite this article**: Chirathivat, N. *et al.* Hemispheric dominance underlying the neural substrate for learned vocalizations develops with experience. *Sci. Rep.* **5**, 11359; doi: 10.1038/srep11359 (2015).

## Figures and Tables

**Figure 1:**
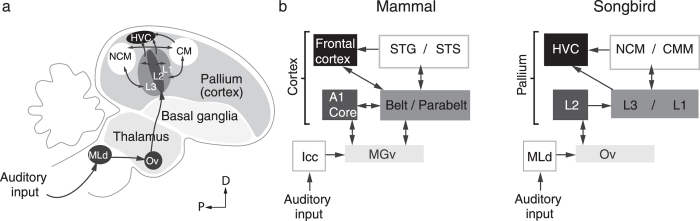
Parallels between avian and mammalian auditory systems. **a**) Simplified diagram (not to scale) of the avian auditory system. The ascending auditory pathway (dark grey arrows) includes a midbrain nucleus (MLd), a thalamic nucleus (Ov) and several cortical regions. The primary auditory cortex (Field L2) connects to the secondary auditory regions Field L3 and L1, which in turn are reciprocally connected to regions analogous to the mammalian auditory association cortex (NCM and medial and lateral CM). (**b**) Block diagram highlighting parallels between mammalian and songbird circuitry. Adapted from^63^,^64^,^65^,^66^,^67^.
